# Supplementary material for: Residue-Specific Epitope Mapping of the PD-1/Nivolumab Interaction Using X-ray Footprinting Mass Spectrometry
Source: Antibodies (Basel). 2024 Sep 19;13(3):77. doi: 10.3390/antib13030077 (PMC11417893; doi:10.3390/antib13030077)
Supplement: Supplementary file 1 [file antibodies-13-00077-s001.zip › antibodies-3020147-supplementary.pdf]

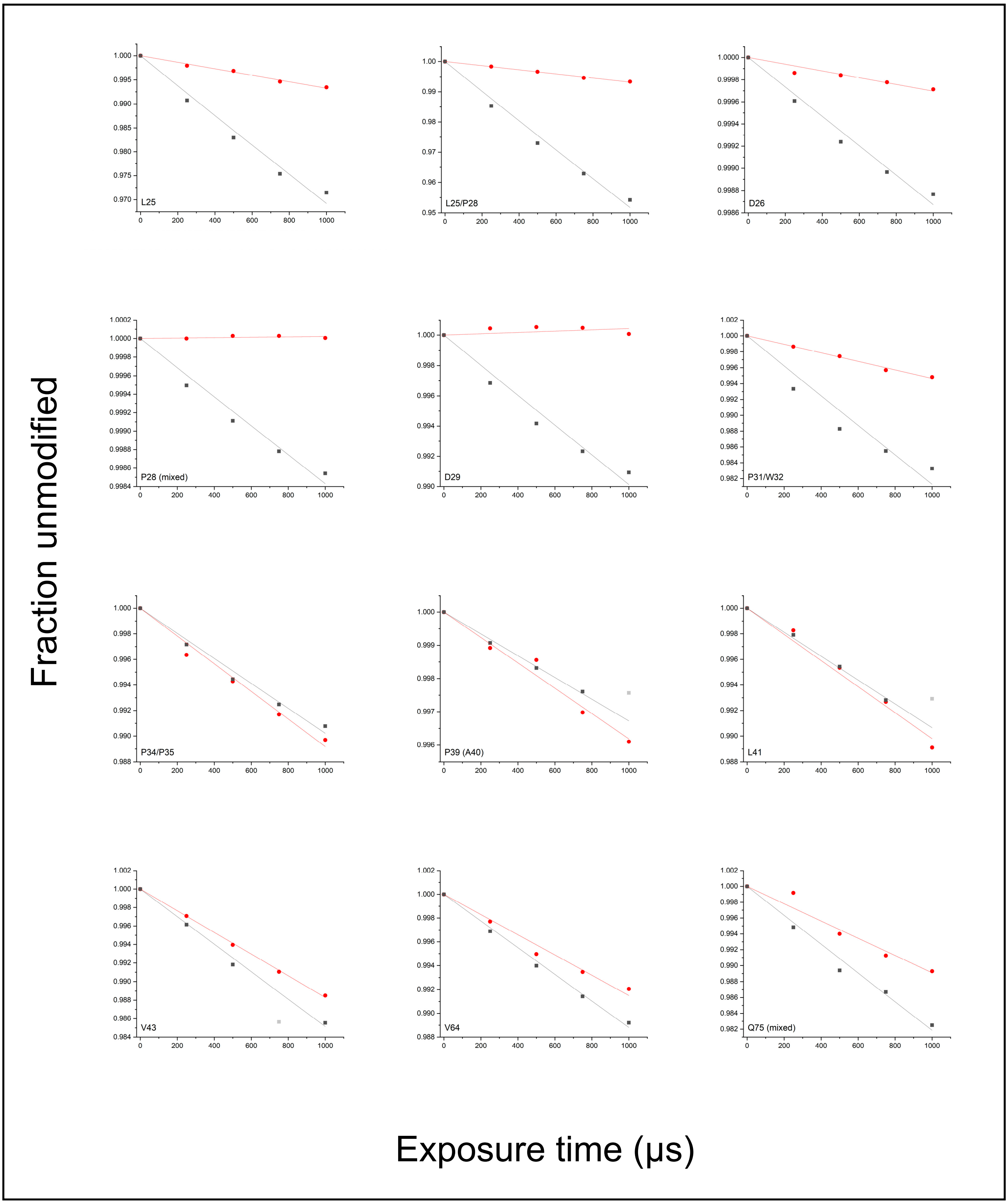

L25
L25/P28
D26
P28 (mixed)
D29
P31/W32
P34/P35
P39 (A40)
L41
V43
V64
Q75 (mixed)
Fraction unmodified
Exposure time (μs)

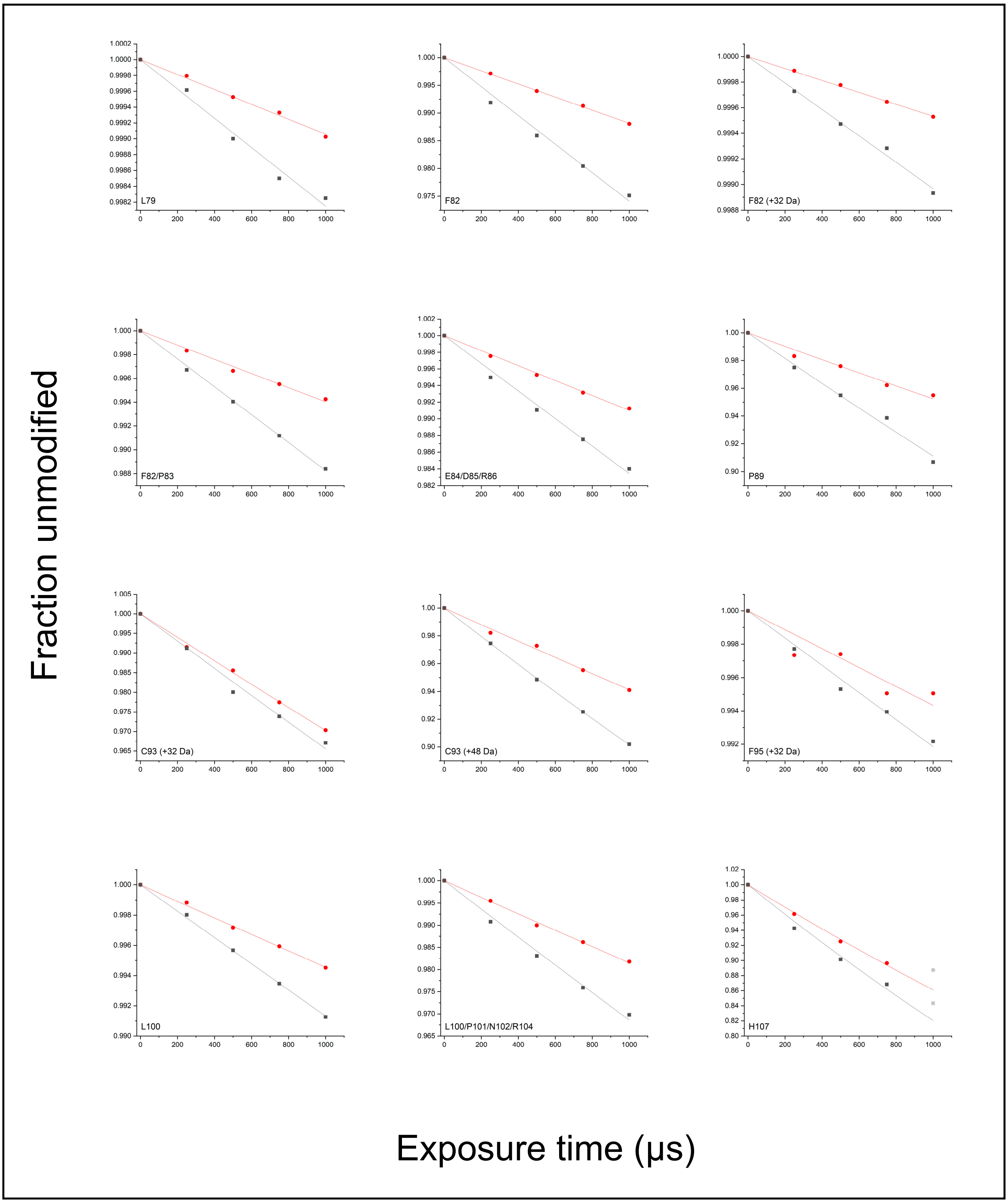

Fraction unmodified
L79
F82
F82 (+32 Da)
F82/P83
E84/D85/R86
P89
C93 (+32 Da)
C93 (+48 Da)
F95 (+32 Da)
L100
L100/P101/N102/R104
H107
Exposure time (μs)

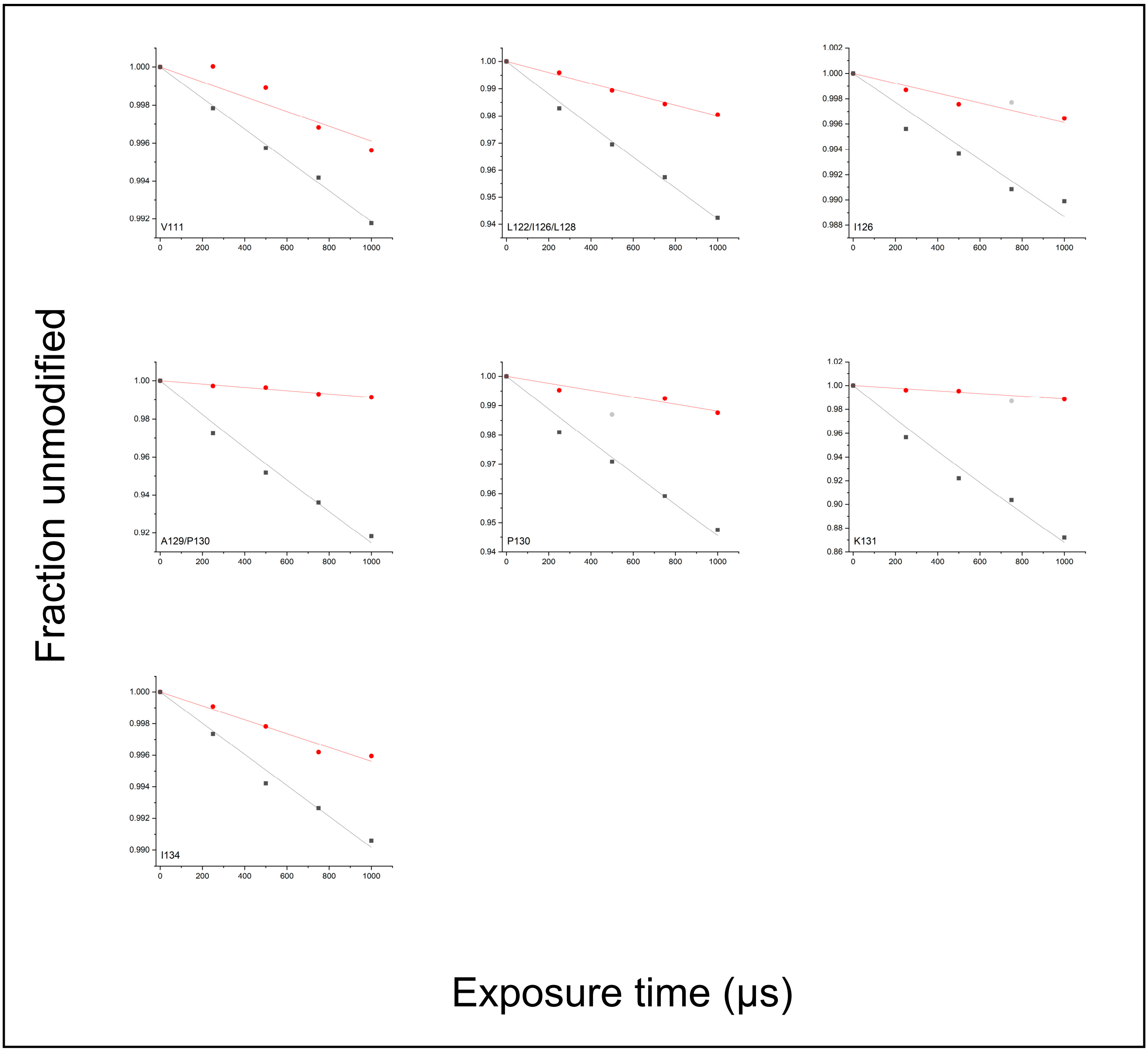

Figure S1. **Residue-specific dose response plots**. Black-square fraction unmodified data points represent free PD-1 while red-circle data points represent the PD-1/nivolumab complex. The solid line represents a first-order exponential fit to determine the hydroxyl radical reactivity rate constant, *k*(s^{-1}).

Table S1. XFMS hydroxyl radical reactivity rate constants.

Seq. #	Sequence[a]	Modified residue[b] (+16 Da)	Hydroxyl radical reactivity rate constant[c], k (s^{-1})		R[d] (k_{PD-1} / $k_{Complex}$)
			k_{PD-1} (s^{-1})	$k_{Complex}$ (s^{-1})	
25-32	LDSPDRPW	L25	31.28 ± 1.28	6.77 ± 0.21	4.62 ± 0.24
		L25/P28	49.47 ± 1.66	6.85 ± 0.12	7.23 ± 0.27
		D26	1.33 ± 0.06	0.30 ± 0.02	4.38 ± 0.40
		P28 (mixed)	1.57 ± 0.07	0.02 ± 0.00	72.67 ± 3.29
		D29	9.92 ± 0.51	0.43 ± 0.00	23.05 ± 1.19
		P31/W32	18.90 ± 1.35	5.35 ± 0.13	3.53 ± 0.27
33-42	NPPTFSPALL	P34/P35	9.83 ± 0.35	10.88 ± 0.41	0.90 ± 0.05
		P39 (A40)	3.28 ± 0.08	3.82 ± 0.19	0.86 ± 0.05
		L41	9.39 ± 0.21	10.26 ± 0.45	0.92 ± 0.05
43-52	VVTEGDNATF	V43	14.96 ± 0.42	11.77 ± 0.12	1.27 ± 0.04
64-68	VLNWY	V64	11.26 ± 0.24	8.53 ± 0.36	1.32 ± 0.06
70-78	MSPSNQTDK	Q75 (mixed)	18.31 ± 0.65	10.96 ± 0.77	1.67 ± 0.13
79-86	LAAFPEDR	L79	1.85 ± 0.07	0.94 ± 0.02	1.97 ± 0.08
		F82	26.23 ± 0.80	11.91 ± 0.09	2.20 ± 0.07
		F82 (+32 Da)	1.03 ± 0.03	0.47 ± 0.00	2.21 ± 0.06
		F82/P83	11.80 ± 0.14	6.00 ± 0.17	1.97 ± 0.06
		E84/D85/R86	16.71 ± 0.45	9.04 ± 0.14	1.85 ± 0.06
87-94	SQPGQDCR	P89	92.99 ± 2.99	48.82 ± 2.14	1.90 ± 0.10
		C93 (+48 Da)	103.62 ± 0.37	60.43 ± 1.50	1.71 ± 0.04
80-95	AAFPEDRSQPGQDCRF	C93 (+32 Da)	35.03 ± 1.14	30.20 ± 0.43	1.16 ± 0.04
		F95 (+32 Da)	8.18 ± 0.27	5.68 ± 0.59	1.44 ± 0.16
97-104	VTQLPNGR	L100	8.72 ± 0.08	5.47 ± 0.08	1.60 ± 0.03
		L100/P101/N102/R104	31.91 ± 0.79	18.67 ± 0.30	1.71 ± 0.05
105-112	DFHMSVVR	H107	197.97 ± 8.44	149.70 ± 2.81	1.32 ± 0.06
		V111	8.17 ± 0.14	3.90 ± 0.53	2.09 ± 0.30

116-131	NDSGTYLCGAISLAPK	L122/I126/L128	59.68 ± 1.10	20.18 ± 0.50	2.96 ± 0.09
		A129/P130	89.12 ± 3.14	8.79 ± 0.40	10.14 ± 0.59
122-128	LCGAISL	I126	11.38 ± 0.80	3.88 ± 0.34	2.93 ± 0.34
129-138	APKAQIKESL	P130	55.98 ± 2.17	11.93 ± 1.04	4.69 ± 0.46
		K131	141.65 ± 5.79	11.36 ± 0.75	12.47 ± 0.98
132-135	AQIK	I134	9.89 ± 0.36	4.39 ± 0.24	2.25 ± 0.15

[a] Peptide sequences of chymotryptic and tryptic fragments used for identification and quantification of modification sites

[b] Sites of modification were localized based on LC-MS/MS. Multi-site notation indicates reduced peak resolution due to co-elution of +16 Da peptide isomers.

[c] The residue-level hydroxyl radical reactivity rate constant was determined from the first-order exponential fit of the dose response plot of fraction unmodified as a function of exposure time. Error estimates represent the SE of the curve fit.

[d] The ratio (R) of hydroxyl radical reactivity rate constants represents a fold decrease (>1) or increase (<1) in the solvent accessibility of the modified residue(s). Error estimates represent the SD of the ratio.
